# Untreated, uncontrolled and below-target hypertension in southern Africa: a population-based prevalence and care cascade assessment in rural Lesotho

**DOI:** 10.1136/bmjgh-2025-020344

**Published:** 2026-02-27

**Authors:** Iliana M Esquivel-Valdés, Giuliana Sanchez-Samaniego, Ravi Gupta, Thesar Tahirsylaj, Fabian Raeber, Mamakhala Chitja, Malebona Mathulise, Thuso Kabi, Mosoetsi Mokaeane, Malehloa Maphenchane, Molulela Manthabiseng, Makhebe Khomolishoele, Mota Mota, Sesale Masike, Matumaole Bane, Mamoronts’sane P Sematle, Retselisitsoe Makabateng, Lebohang Sao, Mosa Tlahani, Pauline Grimm, Thilo Burkard, Frédérique Chammartin, Alain Amstutz, Felix Gerber, Niklaus Daniel Labhardt

**Affiliations:** 1 Division of Clinical Epidemiology, Department of Clinical Research, University Hospital Basel, Basel, Switzerland; 2 University of Basel, Basel, Switzerland; 3 SolidarMed Lesotho, Maseru, Lesotho; 4 Ministry of Health Lesotho, Maseru, Lesotho; 5 SolidarMed Switzerland, Lucerne, Switzerland; 6 Department of Cardiology, University Hospital Basel, Basel, Switzerland; 7 Medical Outpatient Department and Hypertension Clinic, ESH Hypertension Centre of Excellence, University Hospital Basel, Basel, Switzerland

**Keywords:** Hypertension, Treatment, Africa South of the Sahara

## Abstract

**Introduction:**

Hypertension programmes have expanded substantially in low-income and middle-income countries, yet treatment and control rates remain insufficient. Programme scale-up may lead to suboptimal health outcomes and resource allocation if diagnostic accuracy, monitoring and treatment protocol adherence are inadequate. This study aimed to estimate the prevalence of untreated, uncontrolled and below-target hypertension in rural Lesotho, and to identify factors associated with each condition.

**Methods:**

We conducted a population-based cross-sectional study nested within the Community-Based Chronic Care Lesotho (ComBaCaL) cohort study (NCT05596773). Adult cohort participants ≥18 years were eligible for home-based standardised blood pressure (BP) measurement. Hypertension was defined by averaged elevated BP measurements or current use of antihypertensive medication. Uncontrolled hypertension was defined as on-treatment BP ≥140/90 mm Hg, and below-target hypertension as on-treatment systolic BP <110 mm Hg. Multivariate regression models were conducted to identify associated factors.

**Results:**

Between 8 September 2023 and 10 February 2025, 8236 adult participants were screened, with 18.3% (n=1505) diagnosed with hypertension. Of those diagnosed, 75.1% (n=1130) were on treatment and 24.9% (n=375) untreated. Among those on treatment, 53.5% (n=605) were controlled, 26.3% uncontrolled (n=297) and 20.2% (n=228) below target. Female sex, age ≥65 years, diabetes and a history of stroke or myocardial infarction were associated with lower odds of being untreated, while smoking and alcohol consumption increased these odds. Taking ≥3 antihypertensive drugs and non-adherence were associated with a higher risk of uncontrolled hypertension. Dual antihypertensive therapy was associated with a lower risk of being below target, while a history of stroke or myocardial infarction increased this risk.

**Conclusions:**

Despite higher-than-expected hypertension treatment and control rates, substantial gaps remain, including untreated, uncontrolled and below-target hypertension, underscoring the need to strengthen diagnostic accuracy, monitoring and adherence to treatment protocols, with particular attention to high-risk groups.

**WHAT IS ALREADY KNOWN ON THIS TOPIC**

- Despite the global improvements in hypertension care observed over the past decades, significant treatment and control gaps remain in low-income and middle-income countries (LMICs).
- While research in LMICs has predominantly focused on underdiagnosis and undertreatment, the issue of blood pressure (BP) levels below treatment target remains insufficiently explored.

**WHAT THIS STUDY ADDS**

- Provides a comprehensive hypertension care cascade assessment in Lesotho by reporting the prevalence of untreated, uncontrolled and below-target hypertension, along with factors associated with these three conditions.
- Reveals instances of potential overtreatment, underscoring the need for accurate diagnosis, regular BP monitoring and tailored treatment strategies to ensure effective hypertension management and avoid potential harm.

**HOW THIS STUDY MIGHT AFFECT RESEARCH, PRACTICE OR POLICY**

- Supports evidence to develop targeted interventions aimed at improving treatment coverage and reducing treatment inertia to achieve optimal control and avoid potential overtreatment.
- Encourages further research to elucidate the issue of overtreatment and the importance of adhering to current recommendations to ensure accurate diagnoses and effective treatment strategies.

## Introduction

 Hypertension is the leading risk factor for cardiovascular diseases and a major cause of mortality and disability, accounting for 10.9 million deaths and 226 million disability-adjusted life years worldwide in 2021.^1 2^ Global treatment coverage increased from 22% in 1990 to 42% in 2019, along with a rise in control rates from 5% to 21% in the same period.^2^ Nonetheless, advancements have been slower in low-income and middle-income countries (LMICs), where treatment and control rates remain disproportionally low.^2^,^4^

While ongoing efforts are required to further improve awareness of hypertension and access to care, potential adverse events that may arise from scale-up of hypertension programmes with insufficient quality control must be considered. Hypertension diagnosis and management is complex, as blood pressure (BP) is influenced by multiple factors and may vary significantly over time. Out-of-office measurements, such as home BP measurements (HBPMs) or 24-hour ambulatory BP monitoring (24h-ABPM), are recommended to ensure an accurate diagnosis. In the absence of these, diagnosis can be confirmed through repeated standardised office BP measurements on separate visit days.^5^,^7^ Unattended office measurements may offer a practical alternative approach to improve the accuracy of BP assessment by reducing the white-coat effect.^8^,^10^ After the establishment of the diagnosis, regular BP monitoring is required to achieve target BP levels through adequate adjustments of lifelong antihypertensive medication.^5^,^7^

In LMICs, where access to gold-standard diagnostics is often limited, diagnosis is primarily based on office BP measurements. However, inadequate measurement techniques and reliance on diagnostic methods with limited accuracy, such as single-office BP measurements, may lead to misdiagnosis due to physiological BP variability, white-coat hypertension or masked hypertension. These issues can, in turn, result in suboptimal hypertension management, including both undertreatment and BP levels below recommended treatment targets.^11^,^15^ Additionally, insufficient follow-up, such as continued reliance on single-office BP measurements and provider treatment inertia, may further impair the quality of BP management, potentially resulting in uncontrolled hypertension or overtreatment and, thus, suboptimal effectiveness and inefficient resource allocation.^15^

Hypertension care cascades describe the sequential steps from hypertension prevalence and diagnosis to treatment initiation and BP control, providing a structured framework to quantify gaps in care delivery and to evaluate the performance and effectiveness of hypertension programmes.^16^ While most studies in LMICs focus on diagnosis, treatment and control gaps, research addressing BP levels below treatment targets remains scarce. This study aimed to assess the prevalence of untreated, uncontrolled and below-target hypertension in rural Lesotho, and to identify factors associated with these three different conditions.

## Methods

### Study design and setting

We conducted a population-based cross-sectional study nested within the Community-Based Chronic Care Lesotho (ComBaCaL) cohort study (NCT05596773) that aims to generate evidence on the evolving chronic disease burden, risk factors of non-communicable chronic diseases (NCDs) and the effectiveness of health service delivery interventions led by community health workers (CHWs) in rural Lesotho. Established in February 2023, the ComBaCaL cohort study includes inhabitants of 103 randomly selected rural villages in Butha-Buthe and Mokhotlong districts in northeastern Lesotho. The cohort’s design, objectives, informed consent procedures and baseline characteristics have been described elsewhere.^17 18^

Lesotho is a small landlocked country in Southern Africa where an under-resourced health system is facing the double burden of an HIV epidemic and an increasing burden of NCDs, including hypertension.^19 20^ A recent population-based survey in Butha-Buthe and Mokhotlong reported a hypertension prevalence of 21.6%, with treatment and control rates of 67.3% and 49%.^20 21^ A 24h-ABPM in a small subsample of this survey indicated a diagnosis overestimation of 20% and underestimation of nearly 8%, when using standard BP measurement techniques (three consecutive measurements using the average of the last two on a single day).^10^

Hypertension care in Lesotho is provided for free at nurse-led primary care facilities or for a minimal fee at physician-led secondary level facilities. While essential antihypertensive drugs of different classes are usually available and provided free of charge, the availability of functional BP machines is often limited. HBPM or 24h-ABPM is not routinely available.

### Study population

Enrolment into the ComBaCaL cohort started on 22 February 2023; all inhabitants of the study villages were invited to participate during home visits. Given the consent refusal of less than 1%, the ComBaCaL cohort study is an almost complete representation of the overall population in the study villages. All cohort participants ≥18 years were eligible for hypertension screening, which began on 8 September 2023. As the ComBaCaL cohort is an open prospective cohort with constant enrolment of new village inhabitants and censoring of participants who move out of the study village, die or withdraw consent, the number of active participants changes over time. In this study, we included all adult participants in the ComBaCaL cohort with complete BP screening until the date of data extraction on 10 February 2025.

### Data collection

The ComBaCaL cohort is managed by trained CHWs residing in the study villages. All data were collected by CHWs during home visits, supervised by nurses and guided by a tailored data collection and clinical decision support (CDS) application installed on a password-protected tablet. The application is based on the Community Health Toolkit, an open-source software designed for community health initiatives to implement customisable clinical decision and data collection workflows.^22^ Workflows implemented for hypertension screening and diagnosis were based on the Lesotho National Standard Treatment Guidelines.^23^ Further details regarding the eligibility criteria and training of CHWs are published elsewhere.^17^ Costs of CHW-led hypertension screening have been reported separately.^24^

For the present study, data were extracted from two different stages of the study: cohort enrolment and hypertension screening. At cohort enrolment, a broad range of sociodemographic and behavioural risk factor data were collected, including age, sex, level of education, anthropometric measurements, self-reported tobacco use and alcohol consumption, physical activity using the International Physical Activity Questionnaire Short Form (IPAQ-SF) and targeted medical history (self-reported or documented in the participant’s health booklet).^25^ A comprehensive set of household characteristics was collected using the Demographic and Health Survey (DHS) to calculate the International Wealth Index (IWI). The IWI facilitates comparisons of household economic status across LMICs. This wealth index operates on a scale from 0 to 100, where 0 represents the lowest housing quality and absence of essential consumer durables, while 100 signifies the highest housing quality.^26 27^ Furthermore, access to medication was assessed by asking whether any household member could not obtain required medication in the year prior to enrolment.

During hypertension screening, data on hypertension history and current antihypertensive treatment were collected. Height and weight were reassessed and corroborated with previously documented measurements, ensuring an updated body mass index (BMI). Current tobacco use, HIV and diabetes status and associated medication were also recorded. Medical history information was based on self-reports and the participant’s personal health booklet when available. BP measurements were conducted using automated BP machines (Omron M3 comfort HEM7131-E) in a sitting position after determining the appropriate cut-off size and after at least 5 min of rest with the back supported and the arm rested.^28^ During the first measurement, the reference arm was identified as the arm with the higher systolic BP (SBP) and used for all subsequent measurements. At each visit, three BP measurements were taken and the average of the last two readings determined the result. In the case of highly elevated BP (≥180/110 mm Hg), a second series of three measurements after 30 min of rest was conducted on the same day. Hypertension was diagnosed based on the following criteria: (1) current use of antihypertensive medication, (2) two elevated averaged BP readings (BP ≥140/90 mm Hg) on two different days or (3) two highly elevated averaged BP readings (≥180/110 mm Hg) on the same day at least 30 min apart. Participants with self-reported history of hypertension in the absence of current antihypertensive medication were classified according to their BP screening outcomes.

### Operational definitions

Participants who reported current use of antihypertensive medication were classified as being on treatment. Untreated hypertension referred to participants who met the diagnostic criteria for hypertension but were not taking antihypertensive medication. Based on international and national guidelines, uncontrolled hypertension was defined as on-treatment SBP ≥140 mm Hg and/or diastolic BP ≥90 mm Hg.^6 23^ Considering the recommended SBP treatment target of 120–129 mm Hg outlined by international guidelines,^5^ findings indicating an increased risk of adverse events with BP lowering <120 mm Hg,^29^ and assuming a mean SBP visit-to-visit variability of 10 mm Hg based on a recent large-scale cohort study,^30^ we defined below-target hypertension as on-treatment SBP <110 mm Hg. Given the cross-sectional design of our study, this definition includes BP levels below the treatment target due to unnecessary antihypertensive medication resulting from a previous misdiagnosis, excessive antihypertensive medication in participants with true hypertension and potential masked hypertension or intraday variation patterns among on-treatment participants.^31 32^ The remaining participants on antihypertensive treatment were classified as controlled.

BMI was classified into underweight (<18.5 kg/m²), normal weight (18.5–24.9 kg/m²), overweight (25–29.9 kg/m²) and obesity (≥30 kg/m²).^33^ Physical activity was ranked as low, moderate or high, following the IPAQ scoring methods.^25^ Participants were classified as living with diabetes if they had a documented diagnosis and/or were currently on antidiabetic treatment. Limited access to medications was defined as any self-reported difficulty in obtaining required medication by at least one household member over the last year, with potential barriers including financial constraints, transportation barriers and unavailability of the medication. Adherence was assessed by asking participants how many days in the past 4 days they had missed any doses of their prescribed antihypertensive medication. Those who reported taking all doses on all 4 days were classified as adherent.

### Statistical analysis

Descriptive analyses were presented as median with IQR for continuous variables and frequency with proportions (%) for categorical variables. We reported adjusted odds ratios with 95% CI derived from a multivariate logistic regression analysis to identify factors associated with untreated hypertension. Adjusted relative risk ratios with 95% CI were estimated using multivariate multinomial logistic regression analysis to assess factors associated with uncontrolled and below-target hypertension among participants on treatment, with controlled hypertension as the reference category. Multicollinearity was assessed through the Variance Inflation Factor (VIF). Data recorded as ‘refused to say’ or ‘unknown’ were classified as missing and dropped from the analyses. Missing data were assumed to be missing at random. Sensitivity analysis using two alternative thresholds of below-target hypertension (SBP <105 and <115 mm Hg) was conducted to assess the robustness of our findings. Data were analysed using the statistical software R, V.4.4.1, 2024.

## Results

**Figure 1:**
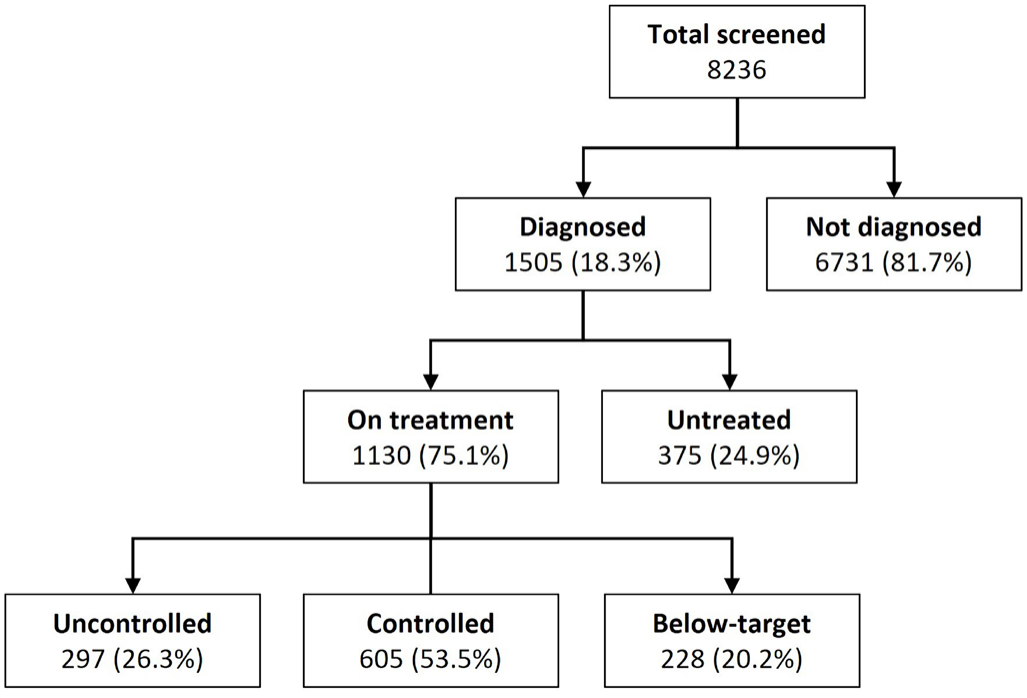
Flow of participants by hypertension diagnosis, treatment and control status.

A total of 9287 adult participants have been enrolled in the ComBaCaL cohort until 10 February 2025. Of these, 8236 had completed hypertension screening and were included in the analysis. The remaining 1051 participants were excluded for the following reasons: 58 died, 114 withdrew consent and 752 moved out of the study villages before completion of the screening; 53 refused BP measurements and 74 had not yet completed the screening at the time of data extraction. Figure 1 displays the flow of participants through the hypertension screening, including diagnosis, treatment and control status.

Table 1 provides the baseline characteristics of the screened population, disaggregated by hypertension diagnosis status, as defined in the Methods section. The overall hypertension prevalence was 18.3% (95% CI 17.5% to 19.1%, 1505/8236), with 9.3% (321/3436) among men and 24.7% (1184/4800) among women. The median age was 40 years (IQR 27–58), with a higher median age among those diagnosed (64 years, IQR 51–74). Around 70% of the participants had no formal education or had only completed primary school. The median IWI score was 30 (IQR 22–39). More than one third were smokers and 18.2% (1500/8236) reported consuming alcohol at least once weekly. The median BMI was 24 kg/m^2^ (IQR 21–28), with a higher proportion of overweight and obesity among diagnosed participants. Nearly three-quarters of the participants reported high levels of physical activity. Overall, 14.6% (1203/8236) of participants reported living with HIV, with no major differences between groups. Diabetes and history of stroke or myocardial infarction prevalences were higher among participants diagnosed with hypertension. Limited access to medications was reported by less than one-fifth of the overall population.

**Table 1:** Baseline characteristics of participants with complete hypertension screening (n=8236) disaggregated by hypertension diagnosis

Characteristic	Not diagnosed, n (%) 6731 (81.7%)	Diagnosed, n (%)* 1505 (18.3%)	Total, n (%) 8236 (100%)
Systolic BP (mm Hg), median (IQR)	113 (105,122)	128 (116,145)	115 (106,125)
Diastolic BP (mm Hg), median (IQR)	75 (69, 80)	84 (76, 94)	76 (70, 82)
Sex			
Male	3115 (46.3%)	321 (21.3%)	3436 (41.7%)
Female	3616 (53.7%)	1184 (78.7%)	4800 (58.3%)
Age (years), median (IQR)	36 (25, 50)	64 (51, 74)	40 (27, 58)
<40	3831 (56.9%)	136 (9.0%)	3967 (48.2%)
40–64	2179 (32.4%)	657 (43.7%)	2836 (34.4%)
≥65	721 (10.7%)	712 (47.3%)	1433 (17.4%)
Education			
No schooling or primary school	4510 (67.0%)	1190 (79.1%)	5700 (69.2%)
Secondary or higher school	2214 (32.9%)	312 (20.7%)	2526 (30.7%)
Refused to say	7 (0.1%)	3 (0.2%)	10 (0.1%)
International Wealth Index, median (IQR)	30 (22, 39)	31 (22, 43)	30 (22, 39)
Q1 (poorest)	1358 (20.2%)	290 (19.3%)	1648 (20.0%)
Q2 (poor)	1376 (20.4%)	271 (18.0%)	1647 (20.0%)
Q3 (middle)	1375 (20.4%)	272 (18.1%)	1647 (20.0%)
Q4 (rich)	1371 (20.4%)	276 (18.3%)	1647 (20.0%)
Q5 (richest)	1251 (18.6%)	396 (26.3%)	1647 (20.0%)
Body mass index (kg/m^2^) median (IQR)	24 (21, 28)	27 (23, 32)	24 (21, 28)
Underweight (<18.5)	413 (6.1%)	62 (4.1%)	475 (5.8%)
Normal weight (18.5–24.9)	3552 (52.8%)	502 (33.4%)	4054 (49.2%)
Overweight (25–29.9)	1670 (24.8%)	451 (30.0%)	2121 (25.8%)
Obesity (≥30)	1039 (15.4%)	488 (32.4%)	1527 (18.5%)
Missing	57 (0.8%)	2 (0.1%)	59 (0.7%)
Smoking			
Current smokers	2473 (36.7%)	455 (30.2%)	2928 (35.6%)
Non-smokers	4250 (63.1%)	1047 (69.6%)	5297 (64.3%)
Refused to say	8 (0.1%)	3 (0.2%)	11 (0.1%)
Alcohol consumption			
1–7 days per week	1255 (18.6%)	245 (16.3%)	1500 (18.2%)
Never or less than 1 day per week	5449 (81.0%)	1254 (83.3%)	6703 (81.4%)
Missing/refused to say	27 (0.4%)	6 (0.4%)	33 (0.4%)
Physical activity			
Low physical activity	771 (11.5%)	275 (18.3%)	1046 (12.7%)
Moderate physical activity	878 (13.0%)	220 (14.6%)	1098 (13.3%)
High physical activity	5022 (74.6%)	997 (66.2%)	6019 (73.1%)
Missing	60 (0.9%)	13 (0.9%)	73 (0.9%)
Living with diabetes	61 (0.9%)	178 (11.8%)	239 (2.9%)
Self-reported HIV status			
Known HIV positive	992 (14.7%)	211 (14.0%)	1203 (14.6%)
Negative ≤12 months ago	3876 (57.6%)	889 (59.1%)	4765 (57.9%)
Negative >12 months ago	988 (14.7%)	251 (16.7%)	1239 (15.0%)
Unknown/refused to say	875 (13.0%)	154 (10.2%)	1029 (12.5%)
Previous stroke or myocardial infarction	27 (0.4%)	84 (5.6%)	111 (1.3%)
Limited access to medications			
No	5556 (82.5%)	1173 (77.9%)	6729 (81.7%)
Yes	1162 (17.3%)	331 (22.0%)	1493 (18.1%)
Unknown/refused to say	13 (0.2%)	1 (0.1%)	14 (0.2%)

* Hypertension was defined as an averaged BP ≥140/90 mm Hg from the last two of three standardised measurements on two separate days; current use of antihypertension medication; or two averaged readings ≥180/110 taken on the same day at least 30 min apart (from the same series of three measurements).

BP, blood pressure.

Table 2 presents the characteristics among participants diagnosed with hypertension (n=1505), disaggregated by treatment status. Overall, 75.1% (95% CI 72.9% to 77.3%, 1130/1505) of participants with hypertension were on treatment, while the remaining 24.9% (95% CI 22.7% to 27.1%, 375/1505) were untreated. Multivariate logistic regression analysis (table 2) shows that female sex, age ≥65 years, diabetes and a history of stroke or myocardial infarction were associated with lower odds of being untreated. In contrast, smoking and alcohol consumption increased the odds of being untreated. The results of the univariate analysis are presented in the online supplemental material (online supplemental table S1).

**Table 2:** Characteristics among participants diagnosed with hypertension (n=1505), disaggregated by treatment status, with multivariate logistic regression for untreated hypertension (n=375)

Characteristic	On treatment, n(%) 1130 (75.1%)	Untreated, n(%) 375 (24.9%)	aOR (95% CI) for untreated hypertension
Systolic BP (mm Hg), median (IQR)	123 (112, 135)	149 (140, 160)	–
Diastolic BP (mm Hg), median (IQR)	80 (73, 87)	97 (92, 103)	–
Sex			
Male	204 (63.6%)	117 (36.4%)	*Reference*
Female	926 (78.2%)	258 (21.8%)	**0.51 (0.37, 0.71**)
Age (years)			
<65	561 (70.7%)	232 (29.3%)	*Reference*
≥65	569 (79.9%)	143 (20.1%)	**0.52 (0.39, 0.71**)
Education (n=1502)			
No schooling or primary school	893 (75.0%)	297 (25.0%)	*Reference*
Secondary or higher school	234 (75.0%)	78 (25.0%)	1.21 (0.85, 1.73)
IWI*			
Q1 (poorest)	208 (69.1%)	93 (30.9%)	1.32 (0.88, 1.99)
Q2 (poor)	224 (74.4%)	77 (25.6%)	0.84 (0.55, 1.28)
Q3 (middle)	221 (73.4%)	80 (26.6%)	*Reference*
Q4 (rich)	231 (76.7%)	70 (23.3%)	0.83 (0.55, 1.27)
Q5 (richest)	246 (81.7%)	55 (18.3%)	0.66 (0.43, 1.03)
Body mass index (kg/m^2^) (n=1503)			
<25	404 (71.6%)	160 (28.4%)	*Reference*
25–29.9	350 (77.6%)	101 (22.4%)	0.91 (0.65, 1.27)
≥30	374 (76.6%)	114 (23.4%)	1.17 (0.84, 1.65)
Smoking (n=1502)			
Non-smokers	835 (79.8%)	212 (20.2%)	*Reference*
Current smokers	293 (64.4%)	162 (35.6%)	**1.90 (1.42, 2.55)**
Alcohol consumption (n=1499)			
Never or less than 1 day per week	980 (78.1%)	274 (21.9%)	*Reference*
1–7 days per week	145 (59.2%)	100 (40.8%)	**1.74 (1.23, 2.46)**
Physical activity (n=1492)			
Low physical activity	208 (75.6%)	67 (24.4%)	*Reference*
Moderate physical activity	169 (76.8%)	51 (23.2%)	0.91 (0.56, 1.48)
High physical activity	744 (74.6%)	253 (25.4%)	0.89 (0.62, 1.28)
Living with diabetes			
No	962 (72.5%)	365 (27.5%)	*Reference*
Yes	168 (94.4%)	10 (5.6%)	**0.23 (0.11, 0.42)**
Living with HIV (n=1351)			
No	866 (76.0%)	274 (24.0%)	*Reference*
Yes	156 (73.9%)	55 (26.1%)	0.85 (0.58, 1.22)
Previous stroke or myocardial infarction			
No	1053 (74.1%)	368 (25.9%)	*Reference*
Yes	77 (91.7%)	7 (8.3%)	**0.19 (0.07, 0.45)**
Limited access to medications (n=1504)			
No	874 (74.5%)	299 (25.5%)	*Reference*
Yes	256 (77.3%)	75 (22.7%)	0.88 (0.62, 1.22)

Percentages were calculated by row. BP values were not included in the logistic regression analysis. A total of 1333 participants with complete covariate data were included in the multivariate model. Variance Inflation Factor was calculated for each predictor, and none exceeded the value of 5.

Bold values indicate statistically significant results.

* IWI quintiles were adjusted for this specific population.

aOR, adjusted odds ratio; BP, blood pressure; IWI, International Wealth Index.

Table 3 shows the characteristics among diagnosed participants on antihypertensive treatment (n=1130), disaggregated by BP control status. Overall, 53.5% (95% CI 50.6% to 56.4%, 605/1130) were controlled, 26.3% (95% CI 23.7% to 28.9%, 297/1130) uncontrolled and 20.2% (95% CI 17.9% to 22.5%, 228/1130) below-target. This implies that among all participants with hypertension: 44.7% (95% CI 42.2% to 47.2%, 672/1505) participants had BP levels above treatment target (untreated or uncontrolled), 40.2% (95% CI 37.7% to 42.7%, 605/1505) within treatment target (controlled) and 15.1% (95% 13.3% to 16.9%, 228/1505) below treatment target. Multivariate multinomial regression analysis (table 3) shows that taking ≥3 antihypertensive drugs and non-adherence to current antihypertensive medication increased the risk of being uncontrolled. Dual antihypertensive therapy decreased the risk of being below target, while a history of stroke or myocardial infarction was associated with an increased risk of below-target hypertension. The results of the univariate analysis are available in the online supplemental material (online supplemental table S2). Sensitivity analyses using alternative SBP thresholds (<105 and <115 mm Hg) are available in the online supplemental material (online supplemental tables S3 and S4). The prevalence of below-target hypertension was 12.2% (95% CI 10.3% to 14.1%, 138/1130) at SBP <105 mm Hg, and 30.2% (95% CI 27.5% to 32.9%, 341/1130) at SBP <115 mm Hg with associated factor estimates remaining consistent across both sensitivity analyses.

**Table 3:** Characteristics among participants diagnosed with hypertension on treatment (n=1130) disaggregated by BP control, with multivariate multinomial logistic regression for uncontrolled (n=297) and below-target (n=228) hypertension, with the controlled group (n=605) as the reference category

Characteristic	Controlled, n (%) 605 (53.5%)	Uncontrolled*		Below-target†	
		N (%) 297 (26.3%)	aRRR (95% CI)	N (%)228 (20.2%)	aRRR (95% CI)
Systolic BP (mm Hg), median (IQR)	122 (117, 128)	145 (137, 155)	–	103 (97, 106)	–
Diastolic BP (mm Hg), median (IQR)	80 (74, 84)	92 (88, 98)	–	71 (66, 76)	–
Sex					
Male	113 (55.4%)	52 (25.5%)	*Reference*	39 (19.1%)	*Reference*
Female	492 (53.1%)	245 (26.5%)	0.90 (0.59, 1.38)	189 (20.4%)	1.24 (0.79, 1.96)
Age (years)					
<65	303 (54.0%)	135 (24.1%)	*Reference*	123 (21.9%)	*Reference*
≥65	302 (53.1%)	162 (28.5%)	1.22 (0.86, 1.71)	105 (18.5%)	0.78 (0.54, 1.13)
Education (n=1127)					
No schooling or primary school	474 (53.1%)	241 (27.0%)	*Reference*	178 (19.9%)	*Reference*
Secondary or higher school	131 (56.0%)	54 (23.1%)	0.79 (0.51, 1.20)	49 (20.9%)	1.05 (0.69, 1.61)
IWI‡					
Q1 (poorest)	108 (47.8%)	72 (31.9%)	1.33 (0.81, 2.18)	46 (20.4%)	1.07 (0.62, 1.83)
Q2 (poor)	117 (51.8%)	63 (27.9%)	1.04 (0.64, 1.69)	46 (20.4%)	0.94 (0.56, 1.58)
Q3 (middle)	124 (54.9%)	56 (24.8%)	*Reference*	46 (20.4%)	*Reference*
Q4 (rich)	121 (53.5%)	56 (24.8%)	0.78 (0.47, 1.29)	49 (21.7%)	1.05 (0.63, 1.74)
Q5 (richest)	135 (59.7%)	50 (22.1%)	0.78 (0.47, 1.29)	41 (18.1%)	0.88 (0.52, 1.50)
Body mass index (kg/m^2^) (n=1128)					
<25	213 (52.7%)	101 (25.0%)	*Reference*	90 (22.3%)	*Reference*
25–29.9	185 (52.9%)	86 (24.6%)	1.17 (0.79, 1.75)	79 (22.6%)	1.05 (0.71, 1.57)
≥30	206 (55.1%)	109 (29.1%)	1.44 (0.96, 2.16)	59 (15.8%)	0.68 (0.44, 1.05)
Smoking (n=1128)					
Non-smokers	458 (54.9%)	208 (24.9%)	*Reference*	169 (20.2%)	*Reference*
Current smokers	146 (49.8%)	89 (30.4%)	1.07 (0.74, 1.55)	58 (19.8%)	1.01 (0.68, 1.51)
Alcohol consumption (n=1125)					
Never or less than 1 day per week	535 (54.6%)	247 (25.2%)	*Reference*	198 (20.2%)	*Reference*
1–7 days per week	66 (45.5%)	49 (33.8%)	1.40 (0.88, 2.24)	30 (20.7%)	1.41 (0.84, 2.35)
Physical activity (n=1121)					
Low physical activity	115 (55.3%)	53 (25.5%)	*Reference*	40 (19.2%)	*Reference*
Moderate physical activity	89 (52.7%)	42 (24.9%)	1.26 (0.72, 2.21)	38 (22.5%)	1.31 (0.74, 2.33)
High physical activity	396 (53.2%)	201 (27.0%)	1.30 (0.85, 2.00)	147 (19.8%)	1.05 (0.66, 1.65)
Living with diabetes					
No	509 (52.9%)	254 (26.4%)	*Reference*	199 (20.7%)	*Reference*
Yes	96 (57.1%)	43 (25.6%)	0.97 (0.62, 1.53)	29 (17.3%)	0.75 (0.45, 1.26)
Living with HIV (n=1022)					
No	471 (54.4%)	217 (25.1%)	*Reference*	178 (20.6%)	*Reference*
Yes	79 (50.6%)	45 (28.8%)	1.55 (0.99, 2.41)	32 (20.5%)	0.91 (0.56, 1.47)
Previous stroke or myocardial infarction					
No	574 (54.5%)	272 (25.8%)	*Reference*	207 (19.7%)	*Reference*
Yes	31 (40.3%)	25 (32.5%)	1.26 (0.67, 2.39)	21 (27.3%)	**1.93 (1.05, 3.55)**
Antihypertensive medication					
Single	314 (53.4%)	134 (22.8%)	*Reference*	140 (23.8%)	*Reference*
Dual	218 (57.2%)	98 (25.7%)	1.09 (0.76, 1.55)	65 (17.1%)	**0.66 (0.46, 0.96**)
≥ 3 drugs	73 (45.3%)	65 (40.4%)	**2.24 (1.45, 3.45**)	23 (14.3%)	0.62 (0.35, 1.08)
Limited access to medications					
No	464 (53.1%)	225 (25.7%)	*Reference*	185 (21.2%)	*Reference*
Yes	141 (55.1%)	72 (28.1%)	1.03 (0.71, 1.49)	43 (16.8%)	0.74 (0.48, 1.12)
Antihypertensive medication adherence (n=1113)					
Adherent	516 (55.8%)	218 (23.6%)	*Reference*	191 (20.6%)	*Reference*
Non-adherent	82 (43.6%)	71 (37.8%)	**2.13 (1.43, 3.17)**	35 (18.6%)	1.26 (0.79, 2.00)

Percentages were calculated by row. BP values were not included in the logistic regression analysis. A total of 994 participants with complete covariate data were included in the multivariate model. Variance Inflation Factor was calculated for each predictor, and none exceeded the value of 5.

Bold values indicate statistically significant results.

* Uncontrolled hypertension defined as on-treatment BP ≥140/90 mm Hg.

† Below-target hypertension defined as on-treatment systolic BP <110 mm Hg.

‡ IWI quintiles were adjusted for this specific population.

aRRR, adjusted relative risk ratio; BP, blood pressure; IWI, International Wealth Index.

## Discussion

We conducted a population-based hypertension prevalence and care cascade assessment among 8236 adults in rural Lesotho. Using home-based standardised BP measurements, the overall hypertension prevalence was 18.3%, with a treatment rate of 75.1% and a corresponding prevalence of untreated hypertension of 24.9%. Among participants on treatment, 53.5% were controlled, 26.3% uncontrolled and 20.2% below target. The observed treatment and control rates align with two previous population-based assessments in Lesotho and a neighbouring rural area in South Africa^17 21 34^; however, these rates are significantly higher than regional modelling estimates.^3^ The discrepancies between real-world and modelling data underscore the need for regular local monitoring of care cascade outcomes as modelled data might be of limited accuracy in times of significant programme changes, such as the intensified scale-up of hypertension diagnosis and treatment.

Nearly half (44.7%) of the participants with hypertension were untreated or had uncontrolled BP levels despite being on treatment. The odds of untreated hypertension were higher among men and participants aged below 65 years compared with women and older ages, consistent with prior research.^35^,^37^ Additionally, participants who smoked or reported alcohol consumption had higher odds of being untreated. These patterns likely reflect a combination of sociocultural norms, behavioural factors and structural health system barriers. Men may have lower engagement with healthcare services, while women are more likely to have regular contact with the health system through maternal or neonatal care programmes.^35 36^ Gender norms that discourage care-seeking and labour migration may further reduce healthcare utilisation among men.^38 39^ Younger adults may perceive themselves at lower risk for chronic diseases and may face additional barriers related to employment and limited clinic accessibility during routine hours.^39 40^ In contrast, older adults may have more frequent contact with the health system due to potential comorbidities and age-related health concerns, which facilitates timely diagnosis and treatment monitoring.^35 39^ Risk-seeking behaviours, such as smoking and alcohol consumption, have been shown in previous studies to be negatively associated with healthcare engagement and adherence to treatment recommendations.^36 41 42^ These findings call for initiatives, such as community-based hypertension screening, linkage or treatment services, to increase awareness and retention in care, addressing underlying risk behaviours, health beliefs and health-seeking patterns.

The risk for uncontrolled hypertension was significantly higher among participants who were taking ≥3 antihypertensive drugs compared with those on monotherapy. Non-adherence to antihypertensive medication was also associated with a higher risk of being uncontrolled. These findings align with recent studies.^43^,^45^ The mechanisms underlying these observations cannot be determined from our analysis. The higher prevalence of uncontrolled BP among individuals prescribed multiple antihypertensive agents may reflect the presence of resistant hypertension, lower adherence related to increased pill burden or a combination of both.^45^,^47^ These findings underscore the need for clinical strategies that systematically assess adherence, consider regimen simplification where feasible, and ensure timely treatment intensification and patient-centred follow-up in the management of hypertension.

One in five participants on antihypertensive medication had SBP below the recommended treatment target, raising concerns about potential overtreatment. Overtreatment is defined as an excess or unnecessary treatment that does not provide any additional benefit and may result in harm due to treatment-related side effects and resource waste.^48^,^50^ Although some studies have acknowledged the issue of overtreatment of hypertension,^51^,^53^ this topic remains insufficiently explored. Overtreatment with antihypertensive medication can increase the risk of hypotension, syncope, hyperkalaemia and acute renal failure.^54^ Overtreatment can be attributed to misdiagnosis and the subsequent prescription of unnecessary treatment, or the excessive use of antihypertensive medication after an accurate diagnosis.^15^ In our study, we were not able to differentiate between these two underlying causes and potential physiological variability, including masked hypertension and intraday BP fluctuations, among on-treatment participants.^31 32^ However, our findings suggest that misdiagnosis may play a major role, given the higher proportion of participants with antihypertensive monotherapy and the lower proportion of classical typical risk factors—such as older age and obesity—observed in the below-target group. The high rate of below-target hypertension could also be explained by physician inertia to reduce or withdraw antihypertensive medication in case of low BP levels.^55^ Using alternative thresholds of ±5 mm Hg for our below-target definition led to approximately 10% change in prevalence in both directions with associated factor estimates remaining consistent in sensitivity analyses, supporting the robustness of our findings.

Accurate and repeated BP measurements are essential for effective hypertension management. In the absence of reliable diagnostic tools, such as 24h-ABPM or HBPM, adherence to standardised recommendations for optimal office BP assessment is essential to reduce the risk of misdiagnosis and guide treatment decisions.^5^,^7^ Failure to confirm persistent elevated or below-target BP may contribute to therapeutic inertia. Repeated BP measurements on separate visits could help differentiate between persistent BP levels below treatment targets and transient low BP readings, in accordance with recommendations by international guidelines.^7 32^ However, this may be challenging in resource-limited settings due to long travel distances and limited access to care, highlighting the need for context-specific, evidence-based recommendations. CHW-led programmes may present a promising solution to enable more regular BP monitoring through home visits.^56^ Both CHW-led and primary care systems may benefit from standardised protocols to ensure accurate diagnoses, promote timely and individualised treatment strategies and strengthen treatment adherence through consistent follow-up and the incorporation of behavioural interventions. Supported by CDS systems, onsite training and close supervision by healthcare professionals, CHWs could facilitate linkage to primary care services, assist with ongoing treatment monitoring, improve access to antihypertensive medication and enable referrals to health facilities for treatment adjustments when BP is persistently elevated or below target.^56 57^ Hypertension guidelines should acknowledge the risk of adverse events and inefficient resource use associated with overtreatment and consider incorporating standardised definitions of lower treatment targets that can be applied in various contexts.

To our knowledge, this study is the first large-scale, population-based assessment of hypertension prevalence and care cascade in southern Africa that includes differentiation of untreated, uncontrolled and below-target hypertension. BP measurements were conducted by trained CHWs following the current standardised BP assessment recommendations. This included a protocol involving three measurements at each visit, with the average of the last two readings considered, and the diagnosis confirmed only after a second elevated BP value on a different day.^58^

Limitations of the study include reliance on self-reported information regarding the current use of antihypertensive medication and relevant comorbidities, which may have introduced recall and social desirability biases. An important contextual consideration is that hypertension care in Lesotho is provided free of charge at nurse-led primary health centres and at minimal cost at secondary-level facilities. Financial barriers to care are therefore relatively limited, and treatment coverage observed in this study may not be directly generalisable to settings where out-of-pocket costs for chronic care are higher. Given the cross-sectional nature of our study, causality cannot be established and differentiation between misdiagnosis, overtreatment and masked hypertension or intraday variation patterns was not possible. Further longitudinal studies, including assessment of treatment patterns over time, are required to elucidate the causes of below-target hypertension. Interventional studies could evaluate CHW-based hypertension programmes in rural settings, exploring how such programmes may impact on treatment patterns and overall BP control.^59^

## Conclusions

While treatment and control rates for hypertension in Lesotho were higher than expected based on available modelling data, a substantial proportion of participants remain untreated or uncontrolled. At the same time, one-fifth of participants on antihypertensive medication showed SBP values below target. Our findings underscore the need for improving access to hypertension screening and care. Furthermore, training of healthcare providers to increase adherence to BP measurement recommendations and reduce inertia to dose adjustments is required to reduce both undertreatment and overtreatment, thereby promoting effective, safe and efficient management of hypertension.

## Supplementary material

10.1136/bmjgh-2025-020344 online supplemental file 1

10.1136/bmjgh-2025-020344 online supplemental table 1

## Data Availability

Data are available upon reasonable request.
