# Supplementary material for: Untreated, uncontrolled and below-target hypertension in southern Africa: a population-based prevalence and care cascade assessment in rural Lesotho
Source: BMJ Glob Health. 2026 Feb 27;11(2):e020344. doi: 10.1136/bmjgh-2025-020344 (PMC12958905; doi:10.1136/bmjgh-2025-020344)
Supplement: online supplemental file 1 [file bmjgh-11-2-s001.docx]

### BMJ Global Health Author Reflexivity Statement

Adapted from Morton, B., Vercueil, A., Masekela, R., Heinz, E., Reimer, L., Saleh, S., Kalinga, C., Seekles, M., Biccard, B., Chakaya, J., Abimbola, S., Obasi, A. and Oriyo, N. (2022), Consensus statement on measures to promote equitable authorship in the publication of research from international partnerships. Anaesthesia, 77: 264-276. <https://doi.org/10.1111/anae.15597>

| **Study conceptualization** | |
| --- | --- |
| 1. How does this study address local research and policy priorities? | The study estimates the prevalence of untreated, uncontrolled, and below-target hypertension in rural Lesotho, a low- and middle-income countries that is facing the double burden of HIV and non-communicable diseases (NCDs) including hypertension. Our findings align with regional health priorities and could inform local strategies to enhance hypertension management. |
| 1. How were local researchers involved in study design? | Local researchers were deeply involved in the study design of the ComBaCaL project by providing critical input to ensure contextual relevance and feasibility. They reviewed and adapted study protocols, contributed to data collection tools, advised on recruitment strategies, and supported identification of relevant outcomes. Through pilot testing and operational planning, they helped optimize procedures and address potential barriers, ensuring that the cohort and nested cross-sectional studies could run smoothly. |
| **Research management** | |
| 1. How has funding been used to support the local research team(s)? | Funding was allocated for local field staff salaries, training workshops, and data management support. |
| **Data acquisition and analysis** | |
| 1. How are research staff who conducted data collection acknowledged? | Community Health Workers (CHWs) are listed in the acknowledgments section and recognized for their valuable contribution to the project. Local nursing assistant who supported data collection are included as co-authors. |
| 1. How have members of the research partnership been provided with access to study data? | All co-authors were provided secure access to anonymized dataset through a shared project database, ensuring transparency and enabling collaborative analysis. |
| 1. How were data used to develop analytical skills within the partnership? | While the analyses were conducted by the lead authors, all co-authors were involved in discussions of analytical approaches and interpretation of results, providing opportunities for collaborative learning. |
| **Data interpretation** | |
| 1. How have research partners collaborated in interpreting study data? | Local co-authors contributed to interpretation of findings by providing context-specific feedback and suggestions on the presentation of results. |
| **Drafting and revising for intellectual content** | |
| 1. How were research partners supported to develop writing skills? | All co-authors actively contributed to manuscript revisions by providing input, feedback, and suggestions on interpretation and presentation of results. |
| 1. How will research products be shared to address local needs? | Findings will be shared with stakeholders at health facility, district and national level to inform about potential strategies for improving hypertension management of the rural population in Lesotho. |
| **Authorship** | |
| 1. How is the leadership, contribution and ownership of this work by LMIC researchers recognized within the authorship? | Local members of the ComBaCaL project team are included as co-authors, recognizing their valuable contribution to the project including study set-up, data monitoring, contextual input, and manuscript review. |
| 1. How have early career researchers across the partnership been included within the authorship team? | This research was completed as the master’s thesis of the first author, providing them, as an early career researcher, with valuable initial experience in conducting research and contributing to academic publishing. |
| 1. How has gender balance been addressed within the authorship? | Gender balance was considered important from the outset when setting up the study team and has been reflected in the authorship, with the first author being female. |
| **Training** | |
| 1. How has the project contributed to training of LMIC researchers? | The local research team was supported through mentorship and hands-on experience across multiple aspects of the study, including study conception, data collection and management, data analysis, and manuscript preparation. |
| **Infrastructure** | |
| 1. How has the project contributed to improvements in local infrastructure? | The project supported local data management systems and facilitated electronic data collection using the community Health Toolkit (CHT), an open-source software designed to implement clinical decision and data collection workflows. Health facilities received support, including training, mentorship, as well as equipment and commodities for hypertension and diabetes management (BP machines, glucometers and strips, medications). The local research team have access to study laptops and all necessary resources to support project implementation. |
| **Governance** | |
| 1. What safeguarding procedures were used to protect local study participants and researchers? | Participant confidentiality was maintained through anonymized data collection, using password-protected tables and data uploaded to secure servers with limited access by the study team. Local staff were trained in ethical conduct and data protection procedures. |
