# Supplementary material for: Untreated, uncontrolled and below-target hypertension in southern Africa: a population-based prevalence and care cascade assessment in rural Lesotho
Source: BMJ Glob Health. 2026 Feb 27;11(2):e020344. doi: 10.1136/bmjgh-2025-020344 (PMC12958905; doi:10.1136/bmjgh-2025-020344)
Supplement: online supplemental table 1 [file bmjgh-11-2-s002.docx]

| **Characteristic** | **Untreated hypertension** (n=375) | |
| --- | --- | --- |
|  | **OR (95% CI)** | **aOR (95% CI)** |
| **Sex** |  |  |
| Male | *Reference* | *Reference* |
| Female | **0.49 (0.37, 0.63)** | **0.51 (0.37, 0.71)** |
| **Age (years)** |  |  |
| <65 | *Reference* | *Reference* |
| ≥65 | **0.61 (0.48, 0.77)** | **0.52 (0.39, 0.71)** |
| **Education (n=1502)** |  |  |
| No schooling or primary school | *Reference* | *Reference* |
| Secondary or higher school | 1.00 (0.75, 1.33) | 1.21 (0.85, 1.73) |
| **International Wealth Index (IWI)*** |  |  |
| Q1 (Poorest) | 1.24 (0.87, 1.76) | 1.32 (0.88, 1.99) |
| Q2 (Poor) | 0.95 (0.66, 1.37) | 0.84 (0.55, 1.28) |
| Q3 (Middle) | *Reference* | *Reference* |
| Q4 (Rich) | 0.84 (0.58, 1.21) | 0.83 (0.55, 1.27) |
| Q5 (Richest) | **0.62 (0.42, 0.91)** | 0.66 (0.43, 1.03) |
| **Body Mass Index (kg/m²) (n=1503)** |  |  |
| <25 | *Reference* | *Reference* |
| 25-29.9 | **0.73 (0.55, 0.97)** | 0.91 (0.65, 1.27) |
| ≥30 | 0.77 (0.58, 1.02) | 1.17 (0.84, 1.65) |
| **Smoking (n=1502)** |  |  |
| Non-smokers | *Reference* | *Reference* |
| Current smokers | **2.18 (1.71, 2.78)** | **1.90 (1.42, 2.55)** |
| **Alcohol consumption (n=1499)** |  |  |
| Never or less than 1 day per week | *Reference* | *Reference* |
| 1 to 7 days per week | **2.47 (1.85, 3.29)** | **1.74 (1.23, 2.46)** |
| **Physical activity (n=1492)** |  |  |
| Low physical activity | *Reference* | *Reference* |
| Moderate physical activity | 0.94 (0.62, 1.42) | 0.91 (0.56, 1.48) |
| High physical activity | 1.06 (0.78, 1.45) | 0.89 (0.62, 1.28) |
| **Living with diabetes** |  |  |
| No | *Reference* | *Reference* |
| Yes | **0.16 (0.08, 0.29)** | **0.23 (0.11, 0.42)** |
| **Living with HIV (n=1351)** |  |  |
| No | *Reference* | *Reference* |
| Yes | 1.11 (0.79, 1.55) | 0.85 (0.58, 1.22) |
| **Previous stroke or myocardial infarction** |  |  |
| No | *Reference* | *Reference* |
| Yes | **0.26 (0.11, 0.53)** | **0.19 (0.07, 0.45)** |
| **Limited access to medications (n=1504)** |  |  |
| No | *Reference* | *Reference* |
| Yes | 0.86 (0.64, 1.14) | 0.88 (0.62, 1.22) |
| **IWI quintiles were adjusted for this specific population.* | | |

**Table S1.** Univariate and multivariate logistic regression for untreated hypertension (n=375) among participants diagnosed with hypertension (n=1505). A total of 1333 observations with complete covariate data were included in the multivariate model. Variance inflation factor (VIF) was calculated for each predictor, and none exceed a value of five.

|  | **Univariate model, RRR (95% CI)** | | **Multivariate model, aRRR (95% CI)** | |
| --- | --- | --- | --- | --- |
| **Characteristic** | **Uncontrolled** ^a^ | **Below-target** ^b^ | **Uncontrolled** ^a^ | **Below-target** ^b^ |
| **Sex** |  |  |  |  |
| Male | *Reference* | *Reference* | *Reference* | *Reference* |
| Female | 1.08 (0.75, 1.55) | 1.11 (0.75, 1.66) | 0.90 (0.59, 1.38) | 1.24 (0.79, 1.96) |
| **Age (years)** |  |  |  |  |
| <65 | *Reference* | *Reference* | *Reference* | *Reference* |
| ≥65 | 1.20 (0.91, 1.59) | 0.86 (0.63, 1.16) | 1.22 (0.86, 1.71) | 0.78 (0.54, 1.13) |
| **Education (n=1127)** |  |  |  |  |
| No schooling or primary school | *Reference* | *Reference* | *Reference* | *Reference* |
| Secondary or higher school | 0.81 (0.57, 1.15) | 1.00 (0.69, 1.44) | 0.79 (0.51, 1.20) | 1.05 (0.69, 1.61) |
| **International Wealth Index (IWI)*** |  |  |  |  |
| Q1 (Poorest) | 1.48 (0.96, 2.28) | 1.15 (0.71, 1.86) | 1.33 (0.81, 2.18) | 1.07 (0.62, 1.83) |
| Q2 (Poor) | 1.19 (0.77, 1.85) | 1.06 (0.66, 1.71) | 1.04 (0.64, 1.69) | 0.94 (0.56, 1.58) |
| Q3 (Middle) | *Reference* | *Reference* | *Reference* | *Reference* |
| Q4 (Rich) | 1.02 (0.66, 1.60) | 1.09 (0.68, 1.75) | 0.78 (0.47, 1.29) | 1.05 (0.63, 1.74) |
| Q5 (Richest) | 0.82 (0.52, 1.29) | 0.82 (0.50, 1.33) | 0.78 (0.47, 1.29) | 0.88 (0.52, 1.50) |
| **Body Mass Index (kg/m²) (n=1128)** |  |  |  |  |
| <25 | *Reference* | *Reference* | *Reference* | *Reference* |
| 25-29.9 | 0.98 (0.69, 1.39) | 1.01 (0.70, 1.45) | 1.17 (0.79, 1.75) | 1.05 (0.71, 1.57) |
| ≥30 | 1.12 (0.80, 1.55) | **0.68 (0.46, 0.99)** | 1.44 (0.96, 2.16) | 0.68 (0.44, 1.05) |
| **Smoking (n=1128)** |  |  |  |  |
| Non-smokers | *Reference* | *Reference* | *Reference* | *Reference* |
| Current smokers | 1.34 (0.98, 1.83) | 1.08 (0.76, 1.53) | 1.07 (0.74, 1.55) | 1.01 (0.68, 1.51) |
| **Alcohol consumption (n=1125)** |  |  |  |  |
| Never or less than 1 day per week | *Reference* | *Reference* | *Reference* | *Reference* |
| 1 to 7 days per week | **1.61 (1.08, 2.40)** | 1.23 (0.77, 1.95) | 1.40 (0.88, 2.24) | 1.41 (0.84, 2.35) |
| **Physical activity (n=1121)** |  |  |  |  |
| Low physical activity | *Reference* | *Reference* | *Reference* | *Reference* |
| Moderate physical activity | 1.02 (0.63, 1.67) | 1.23 (0.73, 2.07) | 1.26 (0.72, 2.21) | 1.31 (0.74, 2.33) |
| High physical activity | 1.10 (0.76, 1.59) | 1.07 (0.71, 1.60) | 1.30 (0.85, 2.00) | 1.05 (0.66, 1.65) |
| **Living with diabetes** |  |  |  |  |
| No | *Reference* | *Reference* | *Reference* | *Reference* |
| Yes | 0.90 (0.61, 1.33) | 0.77 (0.49, 1.21) | 0.97 (0.62, 1.53) | 0.75 (0.45, 1.26) |
| **Living with HIV (n=1022)** |  |  |  |  |
| No | *Reference* | *Reference* | *Reference* | *Reference* |
| Yes | 1.24 (0.83, 1.84) | 1.07 (0.69, 1.67) | 1.55 (0.99, 2.41) | 0.91 (0.56, 1.47) |
| **Previous stroke or myocardial infarction** |  |  |  |  |
| No | *Reference* | *Reference* | *Reference* | *Reference* |
| Yes | 1.70 (0.99, 2.94) | **1.88 (1.06, 3.34)** | 1.26 (0.67, 2.39) | **1.93 (1.05, 3.55)** |
| **Antihypertensive medication** |  |  |  |  |
| Single | *Reference* | *Reference* | *Reference* | *Reference* |
| Dual | 1.05 (0.77, 1.44) | **0.67 (0.48, 0.94)** | 1.09 (0.76, 1.55) | **0.66 (0.46, 0.96)** |
| ≥ 3 drugs | **2.09 (1.41, 3.08)** | 0.71 (0.42, 1.18) | **2.44 (1.45, 3.45)** | 0.62 (0.35, 1.08) |
| **Limited access to medications** |  |  |  |  |
| No | *Reference* | *Reference* | *Reference* | *Reference* |
| Yes | 1.05 (0.76, 1.46) | 0.76 (0.52, 1.12) | 1.03 (0.71, 1.49) | 0.74 (0.48, 1.12) |
| **Antihypertensive medication adherence (n=1113)** |  |  |  |  |
| Adherent | *Reference* | *Reference* | *Reference* | *Reference* |
| Non-adherent | **2.05 (1.44, 2.92)** | 1.15 (0.75, 1.77) | **2.13 (1.43, 3.17)** | 1.26 (0.79, 2.00) |
| **IWI quintiles were adjusted for this specific population.*  ^a^ Uncontrolled hypertension defined as on-treatment blood pressure ≥140/90 mmHg.  ^b^ Below-target hypertension defined as on-treatment systolic blood pressure <110 mmHg. | | | | |

**Table S2.** Univariate and multivariate multinomial logistic regression analysis for uncontrolled (n=297) and below-target (n=228) hypertension among diagnosed participants on treatment (n=1130), with the controlled group (n=605) as the reference. A total of 994 observations with complete covariate data were included in the multivariate model. Variance Inflation Factor (VIF) was calculated for each predictor, and none exceeded the value of five.

|  | **Multivariate model, aRRR (95% CI)** | |
| --- | --- | --- |
| **Characteristic** | **Uncontrolled** ^a^ | **Below-target** ^b^ |
| **Sex** |  |  |
| Male | *Reference* | *Reference* |
| Female | 0.84 (0.55, 1.28) | 0.96 (0.57, 1.62) |
| **Age (years)** |  |  |
| <65 | *Reference* | *Reference* |
| ≥65 | 1.28 (0.92, 1.79) | 0.92 (0.59, 1.42) |
| **Education (n=1127)** |  |  |
| No schooling or primary school | *Reference* | *Reference* |
| Secondary or higher school | 0.81 (0.53, 1.23) | 1.24 (0.76, 2.03) |
| **International Wealth Index (IWI)*** |  |  |
| Q1 (Poorest) | 1.33 (0.82, 2.15) | 1.08 (0.56, 2.05) |
| Q2 (Poor) | 1.04 (0.65, 1.68) | 0.91 (0.49, 1.70) |
| Q3 (Middle) | *Reference* | *Reference* |
| Q4 (Rich) | 0.78 (0.48, 1.26) | 1.02 (0.56, 1.85) |
| Q5 (Richest) | 0.81 (0.49, 1.32) | 1.01 (0.55, 1.87) |
| **Body Mass Index (kg/m²) (n=1128)** |  |  |
| <25 | *Reference* | *Reference* |
| 25-29.9 | 1.16 (0.79, 1.72) | 1.04 (0.65, 1.67) |
| ≥30 | **1.53 (1.03, 2.27)** | 0.76 (0.45, 1.28) |
| **Smoking (n=1128)** |  |  |
| Non-smokers | *Reference* | *Reference* |
| Current smokers | 1.03 (0.72, 1.48) | 0.82 (0.51, 1.33) |
| **Alcohol consumption (n=1125)** |  |  |
| Never or less than 1 day per week | *Reference* | *Reference* |
| 1 to 7 days per week | 1.31 (0.83, 2.05) | 1.19 (0.64, 2.21) |
| **Physical activity (n=1121)** |  |  |
| Low physical activity | *Reference* | *Reference* |
| Moderate physical activity | 1.21 (0.70, 2.09) | 1.22 (0.62, 2.40) |
| High physical activity | 1.29 (0.85, 1.97) | 1.03 (0.60, 1.76) |
| **Living with diabetes** |  |  |
| No | *Reference* | *Reference* |
| Yes | 1.00 (0.64, 1.55) | 0.76 (0.41, 1.40) |
| **Living with HIV (n=1022)** |  |  |
| No | *Reference* | *Reference* |
| Yes | **1.58 (1.02, 2.44)** | 0.97 (0.55, 1.71) |
| **Previous stroke or myocardial infarction** |  |  |
| No | *Reference* | *Reference* |
| Yes | 1.23 (0.66, 2.29) | **2.40 (1.25, 4.63)** |
| **Antihypertensive medication** |  |  |
| Single | *Reference* | *Reference* |
| Dual | 1.16 (0.82, 1.63) | 0.74 (0.48, 1.15) |
| ≥ 3 drugs | **2.41 (1.57, 3.68)** | 0.72 (0.37, 1.38) |
| **Limited access to medications** |  |  |
| No | *Reference* | *Reference* |
| Yes | 1.08 (0.75, 1.56) | 0.86 (0.53, 1.40) |
| **Antihypertensive medication adherence (n=1113)** |  |  |
| Adherent | *Reference* | *Reference* |
| Non-adherent | **2.03 (1.38, 2.99)** | 1.15 (0.66, 1.99) |
| **IWI quintiles were adjusted for this specific population.*  ^a^ Uncontrolled hypertension defined as on-treatment blood pressure ≥140/90 mmHg.  ^b^ Below-target hypertension defined as on-treatment systolic blood pressure <105 mmHg. | | |

**Table S3. Sensitivity analysis:** Univariate and multivariate multinomial logistic regression analysis for uncontrolled (n=297) and below-target (n=138) hypertension among diagnosed participants on treatment (n=1130), with the controlled group (n=695) as the reference. A total of 994 observations with complete covariate data were included in the multivariate model. Variance Inflation Factor (VIF) was calculated for each predictor, and none exceeded the value of five.

|  | **Multivariate model, aRRR (95% CI)** | |
| --- | --- | --- |
| **Characteristic** | **Uncontrolled** ^a^ | **Below-target** ^b^ |
| **Sex** |  |  |
| Male | *Reference* | *Reference* |
| Female | 0.95 (0.61, 1.47) | 1.32 (0.87, 1.99) |
| **Age (years)** |  |  |
| <65 | *Reference* | *Reference* |
| ≥65 | 1.19 (0.83, 1.70) | 0.80 (0.57, 1.12) |
| **Education (n=1127)** |  |  |
| No schooling or primary school | *Reference* | *Reference* |
| Secondary or higher school | 0.72 (0.46, 1.11) | 0.82 (0.55, 1.21) |
| **International Wealth Index (IWI)*** |  |  |
| Q1 (Poorest) | 1.36 (0.81, 2.29) | 1.10 (0.67, 1.80) |
| Q2 (Poor) | 0.97 (0.58, 1.60) | 0.81 (0.50, 1.31) |
| Q3 (Middle) | *Reference* | *Reference* |
| Q4 (Rich) | 0.72 (0.43, 1.20) | 0.84 (0.53, 1.34) |
| Q5 (Richest) | 0.75 (0.45, 1.26) | 0.84 (0.52, 1.34) |
| **Body Mass Index (kg/m²) (n=1128)** |  |  |
| <25 | *Reference* | *Reference* |
| 25-29.9 | 1.15 (0.76, 1.73) | 0.98 (0.68, 1.42) |
| ≥30 | 1.33 (0.87, 2.02) | **0.63 (0.42, 0.93)** |
| **Smoking (n=1128)** |  |  |
| Non-smokers | *Reference* | *Reference* |
| Current smokers | 1.05 (0.72, 1.54) | 0.97 (0.67, 1.40) |
| **Alcohol consumption (n=1125)** |  |  |
| Never or less than 1 day per week | *Reference* | *Reference* |
| 1 to 7 days per week | 1.25 (0.77, 2.03) | 0.97 (0.59, 1.57) |
| **Physical activity (n=1121)** |  |  |
| Low physical activity | *Reference* | *Reference* |
| Moderate physical activity | 1.22 (0.68, 2.17) | 1.09 (0.64, 1.83) |
| High physical activity | 1.25 (0.80, 1.96) | 0.92 (0.62, 1.38) |
| **Living with diabetes** |  |  |
| No | *Reference* | *Reference* |
| Yes | 0.97 (0.61, 1.54) | 0.83 (0.53, 1.29) |
| **Living with HIV (n=1022)** |  |  |
| No | *Reference* | *Reference* |
| Yes | **1.63 (1.03, 2.59)** | 1.07 (0.69, 1.65) |
| **Previous stroke or myocardial infarction** |  |  |
| No | *Reference* | *Reference* |
| Yes | 1.37 (0.70, 2.68) | **1.88 (1.04, 3.42)** |
| **Antihypertensive medication** |  |  |
| Single | *Reference* | *Reference* |
| Dual | 1.01 (0.70, 1.45) | **0.63 (0.45, 0.88)** |
| ≥ 3 drugs | **2.07 (1.32, 3.25)** | **0.60 (0.37, 0.99)** |
| **Limited access to medications** |  |  |
| No | *Reference* | *Reference* |
| Yes | 0.99 (0.67, 1.45) | 0.74 (0.51, 1.08) |
| **Antihypertensive medication adherence (n=1113)** |  |  |
| Adherent | *Reference* | *Reference* |
| Non-adherent | **2.29 (1.50, 3.50)** | 1.38 (0.89, 2.13) |
| **IWI quintiles were adjusted for this specific population.*  ^a^ Uncontrolled hypertension defined as on-treatment blood pressure ≥140/90 mmHg.  ^b^ Below-target hypertension defined as on-treatment systolic blood pressure <115 mmHg. | | |

**Table S4. Sensitivity analysis:** Univariate and multivariate multinomial logistic regression analysis for uncontrolled (n=297) and below-target (n=341) hypertension among diagnosed participants on treatment (n=1130), with the controlled group (n=492) as the reference. A total of 994 observations with complete covariate data were included in the multivariate model. Variance Inflation Factor (VIF) was calculated for each predictor, and none exceeded the value of five.
